# Risk Propagation Model and Its Simulation of Emergency Logistics Network Based on Material Reliability

**DOI:** 10.3390/ijerph16234677

**Published:** 2019-11-23

**Authors:** Tinggui Chen, Shiwen Wu, Jianjun Yang, Guodong Cong

**Affiliations:** 1 School of Statistics and Mathematics, Zhejiang Gongshang University, Hangzhou 310008, China; 2 School of Management and E-Business, Zhejiang Gongshang University, Hangzhou 310008, China; wushiwen45@163.com; 3 Department of Computer Science and Information Systems, University of North Georgia, Oakwood, GA 30566, USA; Jianjun.Yang@ung.edu; 4 School of Tourism and Urban-Rural Planning, Zhejiang Gongshang University, Hangzhou 310008, China; cgd@mail.zjgsu.edu.cn

**Keywords:** complex network, emergency logistics, risk propagation, SIS model

## Abstract

Emergency logistics plays an important role in the rescue process after sudden disasters. However, in the process of emergency logistics activities, risks may arise due to scheduling problems or insufficient supply of warehouse stocks, resulting in an insufficient rescue capacity. In addition, the risk of emergency logistics is random and may exist in a certain link or throughout the whole rescue process of emergency logistics. Consequently, the disaster site may be invaded by sudden disaster risk due to the lack of necessary material supplies. The entire emergency logistics system may be destroyed and cause even greater losses as well. Based on this phenomenon, this paper introduces reliability factors of materials and combines the complex network theory to build an emergency logistics network and analyze the emergency logistics risk propagation mechanism. This paper firstly builds an emergency logistics network based on complex network theory. Then, it combines the improved epidemic model to analyze the influencing factors of risk propagation in the emergency logistics network. Finally, this paper probes into the emergency logistics risk propagation mechanisms and processes in terms of network type, material reliability, rescue speed, etc. Furthermore, this paper identifies key factors for risk control and proposes countermeasures to further spread risks, thereby reducing the risk to loss of economic life.

## 1. Introduction

In recent years, various sudden disasters have occurred all over the world, such as earthquakes, debris flow, fire hazards and plagues, causing huge losses to local economies and life [1]. After a disaster, a lack of relief materials and uneven relief levels likely result in the intensive outbreaks of disaster risk, bringing a series of challenges to rescue work. Generally, the outbreak of emergency logistics risks is sudden, with a wide range of influence and a long duration [2], which brings great challenges to post-disaster material allocation and management. In particular, the risks are highly communicable, which causes the affected area to expand significantly. Therefore, it is of great significance to study the risk propagation mechanism of emergency logistics for post-disaster reconstruction and material dispatch.

**Figure 1:**
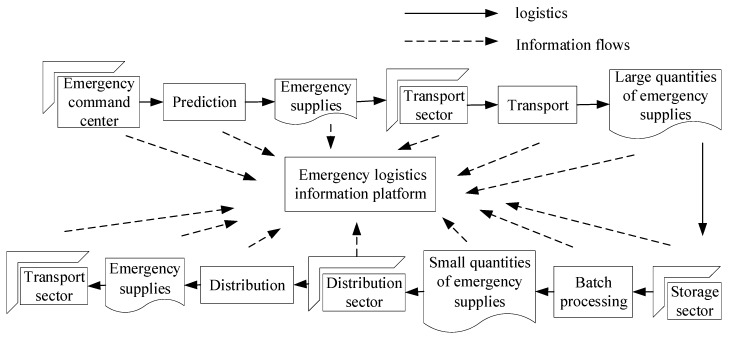
Chained emergency logistics system.

An emergency logistics system is composed of several subsystems, such as an emergency logistics command system, an emergency logistics facilities and equipment system, and an emergency logistics distribution system. Therefore, it is a large and complex system. The integrated management of the whole emergency logistics system is conducive to the restoration of normal social order at the fastest speed after the disaster, similar to a closed chain among emergency logistics subsystems, therefore the operations of emergency logistics are linked, meaning that link or node accidents affect the emergency logistics system rescue process. Risk propagation and diffusion are the main manifestations of this. Apparently, the risk spread throughout the emergency logistics system or area affects the evolution of the other links and nodes. Accordingly, each subsystem or link is the most important role as a collection of people, goods, and materials to reduce sudden disasters, loss, and damage. Therefore, the basis for reducing the loss caused by risks and ensuring the normal operation of the emergency logistics system is the reliability of emergency materials, as shown in Figure 1. The more reliable an emergency supply is, the better it operates. Different material reliability of different links and nodes will form different emergency logistics systems. As a result, in order to ensure the normal operation of the emergency logistics system, the first consideration is to ensure the reliability of emergency materials. Based on the above analysis, it is necessary to study the internal role of the reliability of emergency materials in emergency logistics systems. From the perspective of practical significance, it is feasible and scientific to build the emergency logistics network according to the reliability of the materials, so as to further explore the risk propagation mechanism of emergency logistics in the emergency logistics network.

From the perspective of current studies, scholars rarely conducted theoretical studies on emergency logistics risks. Most relevant research mainly adopted intelligent algorithms to solve problems such as emergency material route scheduling [3] or emergency material distribution site selection [4]. In addition, some literature studied the reliability of complex emergency logistics networks in post-accident rescues [5]. However, very few people considered the role of reliability of emergency materials in the whole emergency logistics system from an overall perspective. Based on this, this paper firstly introduces the reliability factors of emergency materials and constructs the emergency logistics network based on the complex network theory. Following this, the risk propagation process of emergency logistics based on the improved infectious disease model and the evolution rules of emergency logistics from the perspective of the network structure are analyzed. Finally, simulation is performed to testify the effectiveness of the model proposed in this paper and to discuss the influencing factors of risk propagation in order to present the control strategies of emergency logistics risks according to the simulation results.

The structure of this paper is organized as follows: Section 2 is a literature review, and also points out the contributions of this paper, Section 3 constructs the emergency logistics network model and further proposes the improved epidemic model for risk transmission, and Section 4 uses a computer simulation method to analyze the risk propagation mechanism, as well as influencing factors of emergency logistics, and provides some strategies to deal with the risk of sudden disaster in real life. Finally, the conclusions and future research directions are given in Section 5.

## 2. Literature Review

The existence of emergency logistics risk is inevitable, so understanding the mechanism of emergency logistics risk is imperative. Many recent studies were carried out from the perspective of emergency logistics management. For example, Beresford and Pettit [6] proposed that in response to sudden disasters, well-organized communication networks and early warning systems could effectively reduce risks. Jeong et al. [7] proposed an integrated framework of emergency logistics network designed based on efficiency, risk and robustness indicators, and finally verified the applicability of the emergency logistics network constructed through two historical cases. Ji and Zhu [8] introduced emergency supply chain management into disasters, analyzed disaster risks and corresponding countermeasures, put forward a support mechanism for emergency logistics, and finally, verified the conclusion through numerical simulation. Hu and Sheu [9] considered environmental risks, operational risks and the psychological cost for residents and constructed a multi-objective optimization model for emergency logistics, which could minimize the total logistics cost. Li [10] studied the binary language multi-attribute decision-making problem used to evaluate the risk of emergency logistics without complete information and extended the TOPSIS model to solve the problem of emergency logistics risk assessment. Finally, numerical examples were used to illustrate the effectiveness and applicability of the proposed model. Cheng and Yu [11] established an emergency logistics risk assessment system, analyzed the principle and significance of the establishment of the index system, and determined the weight and value of the system by using the fuzzy comprehensive evaluation method and the Delphi method. Finally, the emergency logistics risks (ELR) case of the Yushu earthquake proved that the system could assess the actual risk level. In addition, by studying two cases, Scolobig et al. [12] concluded that disaster risk management required technology development and strengthened communication and cooperation capabilities between different institutions. The above literature mainly concentrated on qualitative analysis, management, and risk control for the risk spreading problem in emergency logistics research, yet quantitative analysis was relatively limited. Because the spread of emergency logistics risk involves many factors, various perspectives should be taken into consideration, which raises the complexity of the problem and difficulty of quantitative analysis and study. Furthermore, risk and reliability always act upon each other, and the study of risk from the perspective of reliability is inevitable and can be quantified through the calculation of the degree of reliability, which is an advantage that qualitative research on risk does not have. Therefore, it is of great practical significance to study the risk propagation of emergency logistics with quantitative reliability. The study better illustrates the mutual relationship between factors influencing the risk propagation of emergency logistics, providing vital importance to expand research in the field of emergency logistics.

In recent years, risk propagation theory has been widely applied in various fields, and relevant research outcomes are very valued for reference. The risk propagation theory was first applied to the medical field for studying the spread of viruses [13], but it also has related applications in other fields. For example, in the field of supply chain, Zuo and Chen [14] established a directed weighted network of suppliers and analyzed supplier network risk factors influencing the spread of an infectious disease model and discovered the key point of risk management so as to effectively reduce the risk of damage to the networks of suppliers. Garvey et al. [15] introduced the Bayesian network framework, which was used to measure the target of risk propagation in the supplier network, as well as applicable specific situations. Deng et al. [16] put forward the concept of the risk communication chain, built a model of an urban risk communication chain by introducing the target-risk framework, and analyzed the specific conditions and paths of risk communication. Wu et al. [17] proposed the Susceptible-Infected-Susceptible Cellular Automation (SIS-CA) infectious disease model, based on which they proposed targeted marginal immune disease strategies. Finally, they verified the feasibility of immune disease strategies by using the collaborative network of aircraft manufacturers. Moreover, in the financial field, Su and Ren [18] proposed a risk communication model based on a multi-layer network, proving that this could help understand the risk communication mechanism in the financial field. Chen et al. [19] established a credit risk transmission network model and discussed the influence of different factors on credit risk transmission through theoretical analysis and numerical simulation. In the field of power communication, Qu et al. [20] presented a method to evaluate the risk propagation threshold of a power network, quantified the risk propagation threshold, and verified the effectiveness of the method using the model. Wang et al. [21] paid attention to the fault risk in modern systems and studied its propagation mechanism with different coupled networks. The results showed that the propagation of fault risk depended on the type and structure of the network. Socievole et al. [22] used the N-Intertwined Mean-Field Approximation (NIMFA) method to evaluate the robustness of network on the SIS infectious disease model, and proposed a virus infection rate model, which combined all of the factors that might affect the infection rate and verified the influence of different factors with a simulation experiment. In the field of sociology, Zhang et al. [23] established a dynamic spatio-temporal comprehensive risk assessment model, considered the rumor propagation process under different influencing factors, and analyzed its sensitivity. Qian et al. [24] studied the spread process of rumors on the complex network through the susceptible-infected-recovered (SIR) propagation and diffusion model and explained the three states of the SIR model using the term “ignorance–disseminator–suffocator”. Finally, different influencing factors were analyzed during the spread of rumors. In the field of manufacturing, Wu et al. [25] studied the defect risk spread process in the assembly process, built the defect model in the assembly process with the SIR model, and verified the effectiveness of the model through theoretical and simulation methods. Different fields involving risk problems experienced relatively abundant achievements, but as far as the authors were concerned, there were few risk propagation models used in the field of logistics. Due to the particularity of the emergency logistics risk propagation process, rational and scientific analyses of the characteristics of emergency logistics risk propagation are greatly needed. The application of the appropriate infectious disease model in emergency logistics risk propagation process and the analysis of the influence factors of risk propagation based on its particularity are of great theoretical significance.

To sum up, there have been relatively few studies regarding the evolution rules of emergency logistics networks and risk propagation mechanisms so far, with most of them focusing on the qualitative analysis of risks. In addition, there have been minimal studies on the risk propagation mechanism of emergency logistics combined with epidemic models at the quantitative level. Therefore, this paper firstly considers the impact of the reliability of emergency materials on a logistics network, puts forward a network model based on the reliability of emergency logistics materials, and studies its evolution rules. Secondly, based on the established emergency logistics network, an improved SIS infectious disease model combined with reliability is proposed to study the risk propagation mechanism of emergency logistics. Furthermore, the main factors that affect emergency logistics risk propagation are also analyzed under the actual situation. Finally, according to the experimental simulation results, the strategy of how to further control the risk propagation of emergency logistics is proposed, so as to reduce the loss caused by the risk.

## 3. Problem Description and Model Construction

### 3.1. Problem Description

**Figure 2:**
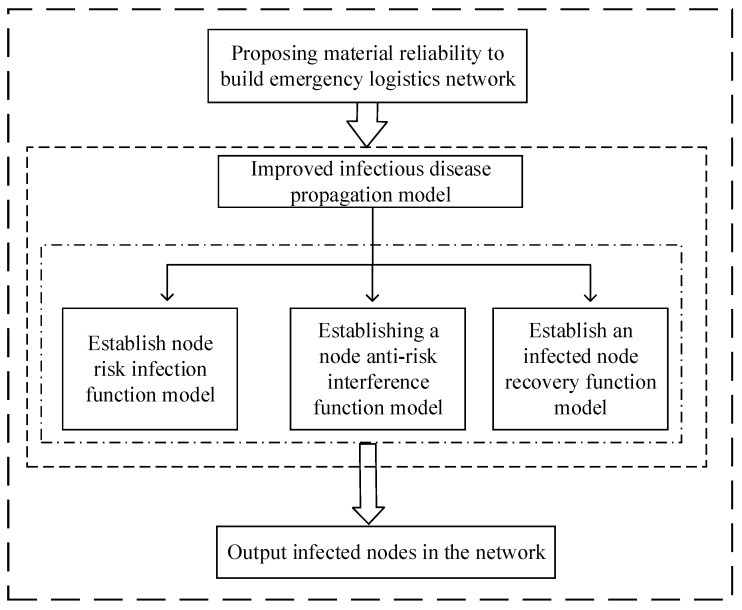
Research framework.

The emergency logistics network was composed of demand nodes generated by sudden disasters in disaster-prone areas. These nodes formed a network according to different risk spread channels. Although analysis of network topology is frequently used, this network could only account for emergency relief logistics issues from the micro level, which was not suitable for describing the emergency logistics risk propagation problem. In the spread of risk, the perceived risk in disaster-prone areas by disaster node is not only acquired by the network connection, but also by different channels. Therefore, combined with the relevant theories of complex network, this paper constructed an emergency logistics risk evolution network and studied the risk propagation mechanism. In the process of emergency logistics risk propagation, this paper integrated the material reliability factors to improve the traditional virus spread model and analyzed the state transition mechanism of demand node in the process of risk propagation and infection, making it more suitable for the study of the emergency logistics risk propagation. The research framework of this paper is shown in Figure 2.

### 3.2. Model Construction

**Figure 3:**
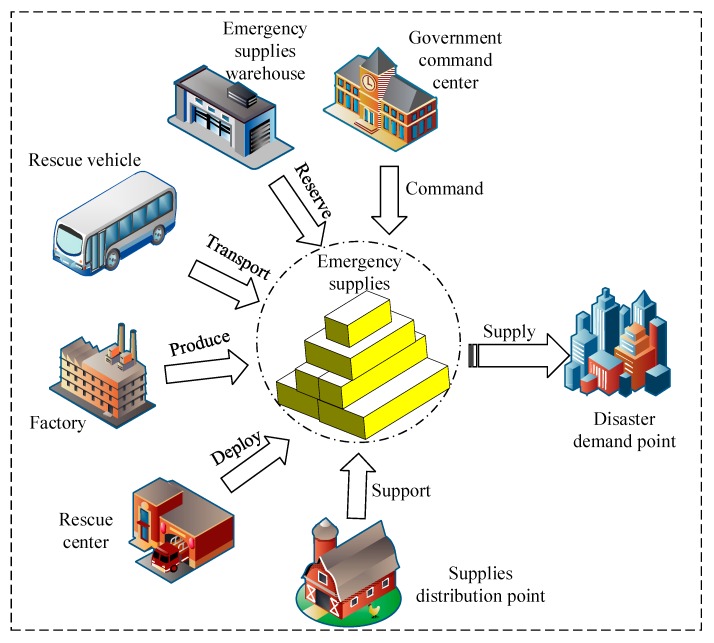
The functions of emergency materials.

Because emergency materials are so important in the emergency logistics system, the reserve and deployment of emergency materials play a significant role in the whole logistics network, especially after sudden disasters [26]. When a sudden disaster occurs, if the node has enough emergency material reservations, it has a strong resistance to the risk. After a sudden disaster, the method and speed of obtaining emergency supplies also affect the risk propagation trend. Emergency materials run through each subsystem of an emergency logistics system and include emergency suppliers, rescue tools, equipment, and transportation. When disaster risk emerges, if there are plenty of relief supplies, full preparation can be made in response to sudden disasters to allow for more rescue time and prevent unnecessary injuries. Therefore, material reliability better explains the related problems of emergency logistics risk propagation, as shown in Figure 3.

This paper combined complex network theory to build an emergency logistics network structure model based on material reliability, which mainly included node growth and priority connection. It should be noted that after an emergency disaster, the node is always be in a risk environment until the end of the disaster, thus the exits the of node and edge were not considered. The specific model construction was performed as follows:

(1) The formation of initial network: Firstly, the emergency logistics network was abstracted into a randomly connected network. In the initial stage, the network was in a small scale, consisting of *m*_0_ demand nodes with random edges. Even edges indicated the possibility of risk propagation between two demand nodes, and vice versa.

(2) Network node connection mechanism: After the outbreak of a sudden disaster, normal nodes in the region became affected nodes as risks arose. It was assumed that the growth of nodes in the network showed a uniform growth trend, and each unit of time increased *m* nodes. The risk propagation of emergency logistics had its unique mechanism, because the connection node was affected by its own state and reliability. This paper mainly studied the evolution mechanism of risk propagation. Therefore, the edges connected the nodes with bigger risks. The probability to be selected for the emergency demand node was related to the node degree and material reliability. As the time changed, the newly demanded nodes were influenced by the existing nodes and connected with the original nodes. Taking the priority connection mechanism in the BA (Barabási & Albert) scale-free network as a reference and considering the characteristics of emergency logistics, it was not possible to grant priority to connection in accordance with the node degree. Here, the connection probability *p*(*i*) was introduced and defined as

(1) $$p(i)\;=\;\theta \frac{k_{i}}{\sum_{i\;=1}^{N}k_{i}}\;+\;(1\;-\;\theta )(1\;-\;\frac{l_{i}}{l_{max}}),$$

where *k_i_* is the degree of existing node *i*, *l_i_* is the reliability level of existing nodes’ material reservation, *l_max_* represents the highest level of material reliability, ξ is the adjustment factor, and *N* is the scale of network nodes. When there was a risk, more external connection channels brought more chances to be infected, including information channels, direct connection channels, and so on. The reliability level of material reservations of nodes also affected the risk of spreading infection. The fewer material reservations, the more likely the emergency logistics network was to be infected by risk. Therefore, the reverse probability was used.

(3) Termination of evolution: When the node size reached a threshold value in advance, evolution terminated.

### 3.3. The Analysis of Emergency Logistics Risk Propagation and Its Rule

Based on emergency logistics network built in Section 3.2, when nodes in the region were affected by sudden disasters and combined with the emergency logistics risk propagation in reality, the risks had the following spread characteristics:

(1) Undirected: When a node was exposed to risks, it generally spread these risks to other connected nodes according to certain rules. Therefore, the study of the emergency logistics network was regarded as an undirected network graph.

(2) Relevant: In an emergency logistics network, each node may not be directly connected, but in the same network there may be indirect links between nodes and the risks may be transmitted to more nodes through other connected nodes in the network. Two nodes without a direct link may also be indirectly connected with other nodes to affect risk spread.

(3) Uncertain: In an emergency logistics network, whether or not nodes communicate risks to each other must be determined. What is the impact of risk propagation intensity? How resistant is it to risk propagation? All these problems lead to the uncertainty of whether neighboring nodes are infected.

Therefore, according to the above analysis of the spread characteristics of emergency logistics, the spread rules were set as follows:

(1) Each node in the emergency logistics network represented the demand node which may have been infected by neighboring nodes, but all had a certain risk resistance capacity. Since the vulnerability of each node and risk value of the connected node were different, these factors had an impact on the incoming and output of risks.

(2) In the process of risk propagation, risks can only be transmitted through two adjacent nodes. Risks could not be spread directly across nodes or unconnected nodes. Nodes were also spread indirectly through intermediate connected nodes.

(3) At time *t*, each node *i* could only be in one of the following two states: infected or susceptible. Node *i* in an infected state at time *t* was denoted as *S_i,t,_* represented by Boolean value 1 and 0, respectively, where 1 represented the infected state and 0 represented the susceptible state.

(4) If some areas suffered from sudden disasters, they generally accepted external assistance. The demand nodes in the risk of infection restored to the susceptible state at a certain probability. However, the susceptible state did not represent a permanent immunity to infection risk, which turned into risk of infection due to the occurrence of secondary disasters, the consumption of materials, and risk irresistibility.

### 3.4. The Risk Propagation Model of Emergency Logistics

At present, traditional epidemic models include SI, SIS, SIR, SIRS (Susceptible-Infected-Removed-Susceptible), etc. The adoption of a particular model depends on the characteristics of the emergency logistics risk propagation. In an emergency logistics network, a node affected by risk may become normal after receiving rescue from the supply node. Therefore, the solo SI epidemic disease model was not suitable to analyze the risk spread of emergency logistics. In addition, once a sudden disaster breaks out, the affected node continues to be influenced by disasters and no immune ones exist. Therefore, the SIR and SIRS models with immune status were not suitable to describe the process of emergency logistics risk propagation. Based on this, the risk propagation model in this paper mainly refers to the susceptible–infected–susceptible (SIS) model. The SIS virus spread model better described the risk propagation of the emergency logistics network, classifies the different states of nodes in the emergency logistics network, and better simulated the risk propagation of the entire emergency logistics network.

The SIS epidemic model is also often used to study virus spread process. According to the above discussion, due to the similarity between emergency logistics risk spread and virus spread, the SIS epidemic model could be used to study the emergency logistics risk propagation process. As long as a sudden disaster occurred, the emergency demand node was in two states: the infection risk state and the susceptible to infection risk state. Similar to virus spread, there were also two states: the infected virus state and the susceptible virus state. As time went on, the material could be exhausted or secondary disasters could occur, causing the risk to continue to spread, so the node became infected by the risk once again. When some emergency demand nodes were in the state of risk infection, they returned to the state of susceptibility to infection by improving their anti-risk ability or obtaining external assistance, that is, the normal state. Generally speaking, the emergency logistics demand node could not absolutely be met by material demands, and there was always a continuous demand for materials, that is to say, it was in the state of susceptibility to infection instead of immune risk. Therefore, the emergency logistics network did not consider the immune node. The two states of nodes in an emergency logistics network are explained below.

**Figure 4:**
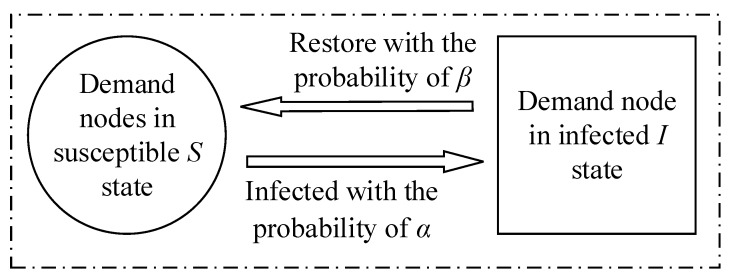
Transformation of node status.

(1) Demand nodes in the susceptible state S: Some demand nodes in the emergency logistics network were in susceptible state. Although such demand nodes were in a healthy state, if they suffered from risk infection under the risk, these nodes were re-transformed into infected nodes with a certain probability.

(2) Demand nodes in the infection state I: Some nodes in the emergency logistics network were in the infection state, which were already affected by the risk and in the risk of infection state. These nodes were likely to spread the infection to other connected nodes. For emergency logistics nodes, due to the shortage of supplies, they were at risk, and the abnormal state was likely to spread to other connected nodes. Based on this, the node state transition in the emergency logistics network was determine, as shown in Figure 4.

According to the above analysis, the risk of emergency logistics was spread on the basis of the emergency logistics network. The symbol descriptions of the risk evolution model are shown in Table 1.

#### 3.4.1. Construction of the Risk Propagation Function

This paper mainly studies issues related to emergency logistics risk propagation. Eigenvector centrality is suitable for describing the long-term influence of nodes and is mainly used for propagation analysis. Therefore, Eigenvector centrality was adopted to evaluate the degree of risk propagation and diffusion of nodes shown as follows:

(2) $$C_{e}\left(i\right){\;=\;\theta }^{-1}\sum_{j=1}^{n}a_{ij}e_{j},$$

where *θ* is the main eigen value of the network adjacency matrix, *a_ij_* is the Boolean variable used to judge connected relations among nodes, and *e_j_* is the corresponding eigenvector.

When risk occurred, the destroying degree was taken into consideration. As a result, *λ* was introduced and used to represent intensity of risk. The greater the *λ* was, the greater the risk was.

According to the above discussion, the risk infection function of nodes in the emergency logistics network was expressed as follows:

ω*_i_* = *λC_e_*(*i*). (3)

#### 3.4.2. Construction of the Anti-Risk Disturbance Function

When risk appeared, due to different material reservations and anti-risk capacity, the closeness centrality between the two nodes was mainly considered. The closer the nodes were, the greater the possibility of infection was. Therefore, the anti-risk disturbance function of node *i* was defined as

(4) $$\tau_{i}\;=\;{kF}_{i}*(1\;-\;k)\frac{l_{i}}{l_{max}},$$

where $\frac{l_{i}}{l_{max}}$ represents the reliability level of material reservation of node *i*, which is used to evaluate the anti-risk ability of demand node when risk occurred. When that happened, the larger the amount of material reservations was, the more reliable it was, and vice versa. In order to facilitate calculation, the ratio of material reservations to the maximum value of nodes was the result of normalization. In addition, *F_i_* is a closeness. In complex networks, the node degree reflected the direct relationship with other nodes. Closeness not only reflected the size of the node degree, but also reflected its position in the network and the global structure of the network. Therefore, the closeness reflected the influence of one node to other nodes through the network. It was suitable to describe the closeness centrality of two nodes in the emergency logistics network as well. The closer the nodes were, the more likely they were to be infected. If there were *N* nodes in the emergency logistics network, the closeness centrality *F(i*) of node *i* was expressed as

(5) $$F_{i}=\frac{1}{\sum_{j=1}^{N}d_{ij}},$$

where *d_ij_* refers to the minimum distance from node *i* to *j.* In order to normalize the closeness, in a network with *N* nodes, the minimum sum of distances from nodes to all other nodes was *min*($\overset{N}{\underset{j=1}{\sum }}d_{1j}$,$\overset{N}{\underset{j=1}{\sum }}d_{2j}$,$\overset{N}{\underset{j=1}{\sum }}d_{3j}$…$\overset{N}{\underset{j=1}{\sum }}d_{Nj}$); therefore, the definition of normalized closeness was

(6) $$F_{i}\;=\frac{\min(\sum_{j=1}^{N}d_{1j},\sum_{j=1}^{N}d_{2j},\sum_{j=1}^{N}d_{3j}\ldots \sum_{j=1}^{N}d_{Nj})}{\sum_{j=1}^{N}d_{1j}}.$$

#### 3.4.3. Construction of the Risk Output Function

In the process of risk output, each emergency logistics demand node usually owns certain anti-risk ability. Since emergency logistics risk propagation is discussed in this paper, the risk infection capacity was set as *ω*; if the risk intensity was greater than the node anti-risk ability, the demand node was affected. If the risk of infection ability was weaker than the anti-risk ability, the demand node would not be subject to interference. The risk output anti-risk infection function was expressed as follows:

(7) $$Q\left(\lambda_{i}\right)=\{\begin{array}{c} 1;\omega \left(i\right)\geq \tau_{i-1}\\ 0;\omega \left(i\right)<\tau_{i-1} \end{array},$$

where *ω*(*i*) is the infected capacity value compared with other nodes in network and *τ_i_*_-1_ means the anti-risk capacity value of *i*’s adjacent node.

#### 3.4.4. Recovery Mechanism from Infected to Susceptible Status

When the emergency logistics risk occurred, some nodes were in the infected status, which may obtain some external non-governmental rescue. Rescue was likely to make the nodes in the infected status convert into a health state that was susceptible to be infected. Combined with the characteristics of emergency logistics and the content mentioned above, in general, the less the demand node of material reservations in the risk status was, the greater the unreliability of the node was, and the greater the possibility of obtaining additional material assistance was. The more channels there were for external communication, the greater the degree of node was, and the greater the possibility of obtaining rescue was. Therefore, according to the above analysis, the transformation from infected to susceptible was expressed as follows:

(8) $$O_{i}=\;k_{i}*(1\;-\;\frac{l_{i}}{l_{max}}),$$

where $\frac{l_{i}}{l_{max}}$ is the reliability of node; if the demand node reserved less materials, the supply nodes gave priority to the nodes with less reservations, that is to say, the possibility of obtaining relief connection was greater. *k_i_* represents the degree of nodes.

### 3.5. Simulation Process of Emergency Logistics Risk

The evolution process of the risk propagation model proposed in this paper was as follows:

Step 1: Build the initial network. The initial network node was *m*_0_. In order to be closer to reality, the nodes in the initial network were connected randomly.

Step 2: Growth mode of network nodes. *m* nodes were added and *m*_0_ nodes were connected to new nodes and the initial network. That is, *m* edges were added every time and the newly added nodes connected to the nodes in the initial network by the selection rule of Equation (1).

Step 3: Return to step 2 to build an undirected network graph with *N* network nodes.

Step 4: Randomly select some nodes in the established network and set them as risk infection node *i*, and set the initial risk propagation intensity as *λ*.

Step 5: During risk propagation, the risk spread to multiple adjacent nodes after transmitting from the risk source, and the infected node recalculated the infection ability according to Equation (3).

Step 6: The risk propagation of adjacent nodes near the infected node *i* in the network calculated the risk resistance of adjacent nodes according to Equation (4), and judged the output risk according to Equation (7).

Step 7: If node *i* was infected at a certain time *t* and became infected, then the state was maintained during *t_d_* thereafter. At the time of *t* + *t_d_* + 1, the infected node calculated the probability *O^i^* according to Equation (8) to eliminate the risk and become the state of susceptible to infection.

Step 8: Repeat step 5–7 until the end of the evolution time.

According to the above evolutionary steps, the number of infected demand nodes in emergency logistics network at time *t, S_i,t_*, was expressed as follows:

(9) $$S_{i,t}{\;=\;S}_{i,t}{\;+\;S}_{{i,t+t}_{d}}Q_{\lambda_{i}}.$$

According to the above analysis, the infection node density *Z*(*t*), which is the ratio of the number of nodes in the emergency logistics network under the state of risk infection to the number of whole network nodes, represented the risk infection density of the emergency logistics network at a certain moment. This was expressed as follows:

(10) $$Z(t)\;=\;\frac{\sum_{i=1}^{N}S_{i,t}}{N},$$

where *N* is the number of whole network nodes.

**Figure 5:**
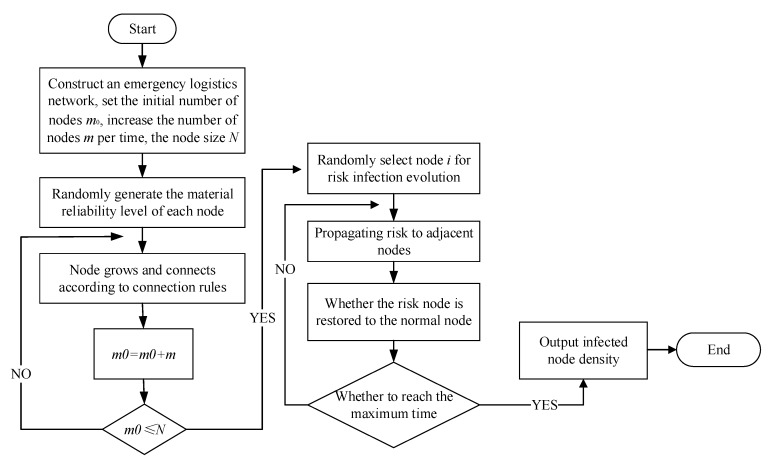
Risk evolution of emergency logistics.

Based on the reliability of emergency material and combined with the complex network theory, an emergency logistics network was built and the risk propagation mechanism was analyzed using the improved SIS model. The evolution process is shown in Figure 5.

## 4. Simulation and Analysis

This section analyzes the impact of network structure, risk level, and rescue speed on the risk propagation of emergency logistics.

### 4.1. Comparative Analysis of Different Network Types

**Figure 6:**
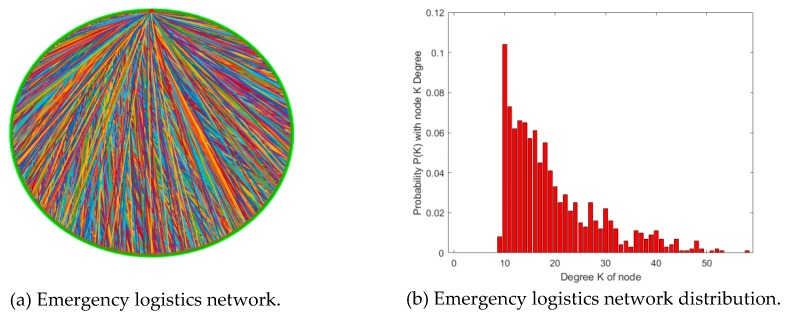
Emergency logistics network sketch map.

In the real world, most networks are not random. Instead, a small number of nodes have a high degree of connection, while most nodes have a low degree of connection. Therefore, most networks in the real world are consistent with scale-free characteristics. Consequently, it is necessary to analyze the characteristics of the emergency logistics network and compare scale-free BA network characteristics similar to the real network [27]. The number of initial network nodes with random edge connections was 10 (*m*_0_ = 10) and the number of each newly added nodes was 10 (*m* = 10), therefore a network was formed with *N* = 1000 nodes. Through evolution, its characteristics were analyzed. The specific results are shown in Figure 6 and Table 2.

According to the above results, the characteristics of the emergency logistics network were basically consistent with power–law distribution. Those with less node degrees occupied the majority, while those with more node degrees occupied less. Among them, the distribution of node degrees was mostly between 10 and 30, and the largest distribution of node degrees was around 50. Due to limited risk propagation channels, the characteristics of this emergency logistics network were basically consistent with the situation in the real world. The topological characteristics of emergency logistics risk also conformed to the scale-free network characteristics. Thus, from the perspective of theoretical data, the constructed emergency logistics network was more reasonable.

**Figure 7:**
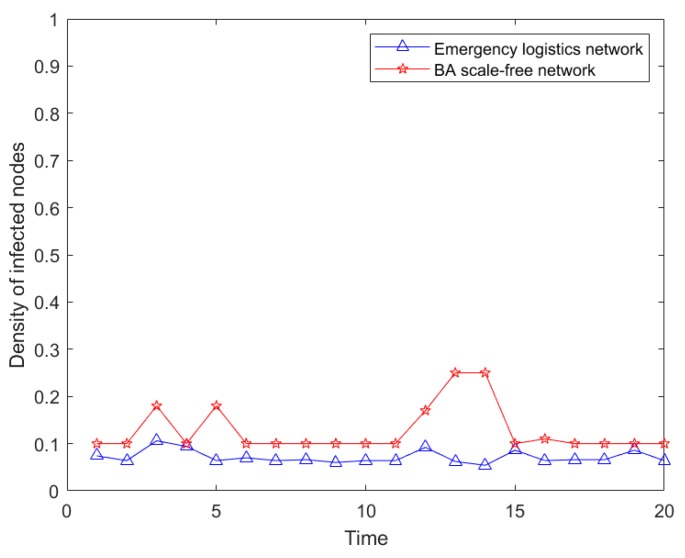
Infection scale of two networks.

Based on the characteristics of emergency logistics network topology and simulation analysis of two different risk propagation methods on the network, the related parameters were set as follows: The network size was *N* = 500, the proportion of the initial infection sources was *Φ* = 0.1, the risk of infection levels was *α* = 0.5, and the recovery capacity was *β* = 0.1. After testing 20 times, the average results were obtained, as shown in Figure 7.

Figure 7 shows that under the same parameters, two kinds of risk propagation density were similar. The infection density of the BA network risk was slightly higher than that of emergency logistics network, and the BA network was more volatile than the emergency logistics network, which had strong stability for risk propagation. It should be specified that the construction of emergency logistics network was more suitable for describing the risk propagation process of the emergency logistics system. Therefore, in the actual situation, when demand nodes suffer from sudden risk, material reliability should be taken into consideration in order to formulate more accurate and appropriate measures so as to avoid emergency logistics network loss. This further demonstrated the effectiveness of the emergency logistics network.

### 4.2. Risk Degree and Recovery Capacity

**Figure 8:**
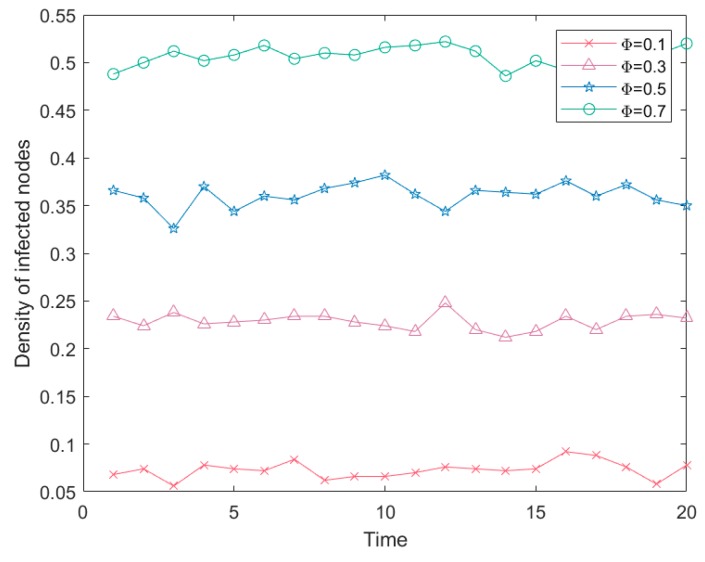
Risk propagation of different initial infection sources.

In the process of studying risk propagation, it is necessary to further inquiry the influence factors of risk propagation so as to provide better strategies for risk control. Therefore, other factors in risk propagation were further simulated in this paper, and the relevant parameters were set as follows: First, a simulation was carried out for different initial infection sources. The network size was set as *N* = 500, the risk infection level was *α* = 0.5, and the recovery capacity was *β* = 0.1. The influence of different initial infection sources on risk infection density is shown in Figure 8:

As can be seen from Figure 8, if the other parameters were kept unchanged, the density of infected nodes increased along with the increase of the proportion of initial infection sources, indicating that the number of initial risk sources affected the number of final risk infections. In the emergency logistics network, if there were more demand nodes infected by risks in the initial stage of risk outbreak, the probability of infecting other connected nodes also increased, and then the final density of infected nodes was higher as well.

**Figure 9:**
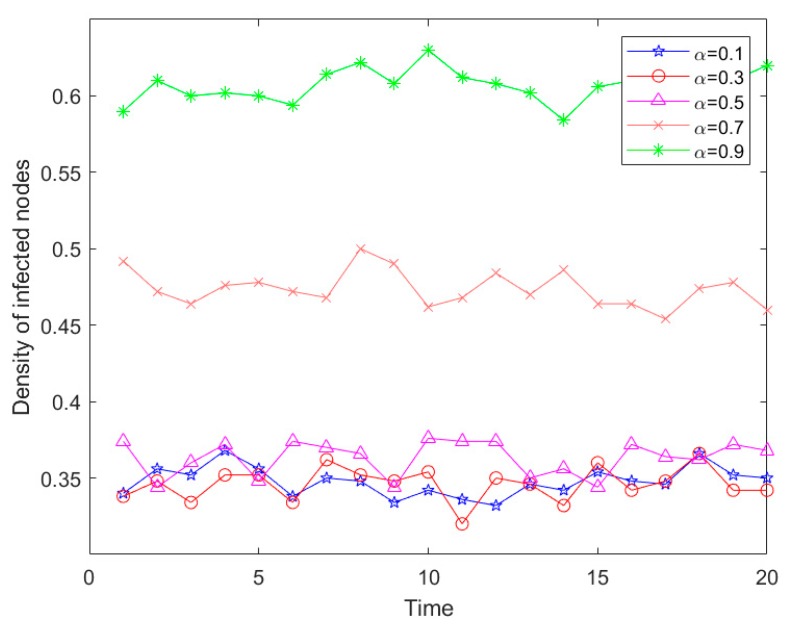
Risk propagation trends of different risk levels.

In addition, the different levels of infection risk and the recovery rate had the potential to impact on risk propagation density. The network scale was set ay *N* = 500, the proportion of the initial infection sources was *Φ* = 0.5, and the recovery rate was *β* = 0.1. The influence of different risk levels for risk propagation density is shown in Figure 9.

As seen in Figure 9, with the increase in risk level, the proportion of infected nodes further increased. When sudden disasters with a strong destructive force broke out, the affected nodes also increased, which was consistent with the reality. However, during the risk transmission process, all risk levels had a small range of fluctuation, particularly with the increase in the risk of infection level, where the fluctuations became slightly larger. In addition, due to the randomness of the material reliability of each node, the material reliability variance of different infected nodes also increased with increased infected nodes, resulting in a larger fluctuation. When the risk infection level was between 0.1 and 0.5, the risk infection proportion varied slightly. This was because it was affected by the Eigenvector index of the nodes, which indicated the influence of nodes. Under the relatively low risk infection level, the final impact was relatively small. In practical emergency logistics, if the radiation force of a node was small, the final loss was relatively small when a small scope of sudden disaster occurred. This observed phenomenon was consistent with the actual situation, hence, the gap of the risk infection ratio was relatively small.

**Figure 10:**
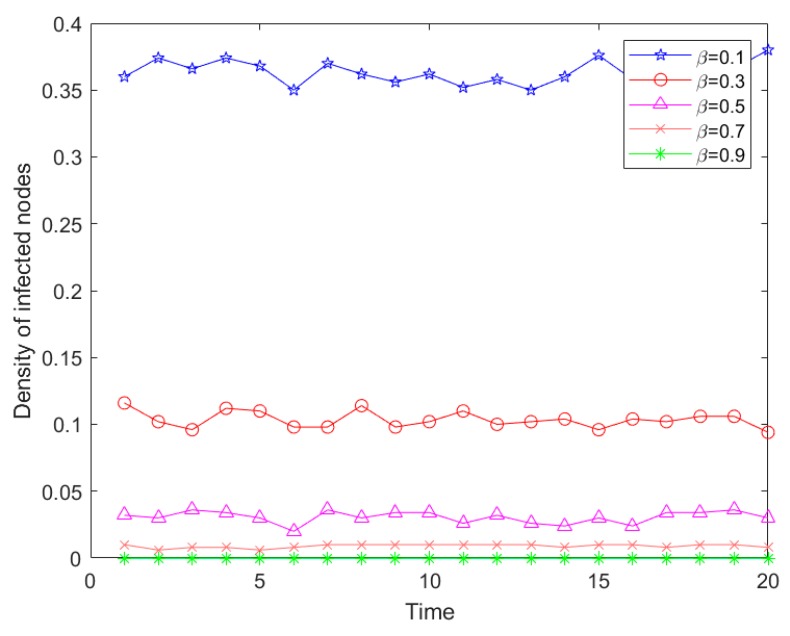
Risk propagation trend of different risk recovery capacity.

When the infection risk level was unchanged, our experiment set the network parameters as *N* = 500, the initial infection sources proportion was *Φ* = 0.5, and the risk rate was *α* = 0.5. The simulation results are shown in Figure 10.

As shown in Figure 10 above, with the improvement of the node’s risk recovery capacity, the density of risk infection in the network decreased, which demonstrated better risk resistance with a smaller range of risk infection. In addition, when the recovery capacity was greater than 0.5, that is, the ratio between recovery capacity and risk infection level was greater than 1, the proportion of node infection in the emergency logistics network was very low, and the risk infection rarely fluctuated. This was because the strong recovery capacity was conducive to the further suppression of risk propagation.

Figure 9 and Figure 10 show that the infection density of risk was high, the infection speed was very fast, and the risk explosion was strong. These two figures also demonstrate that at the beginning of the infection, the infection density of risk fluctuated. Combined with the analysis of emergency logistics risk characteristics, the node degree distribution was between 10 and 30 mostly, and the largest value was 50 or so. Therefore, the initial infection nodes were connected with some nodes with higher degrees. In addition, the fluctuation in infection risk was because the infection of each node was connected to other nodes and the numbers were different, which was consistent with the real situation that the infection method, channel, and connected node were different.

### 4.3. The Influence of Rescue Speed

**Figure 11:**
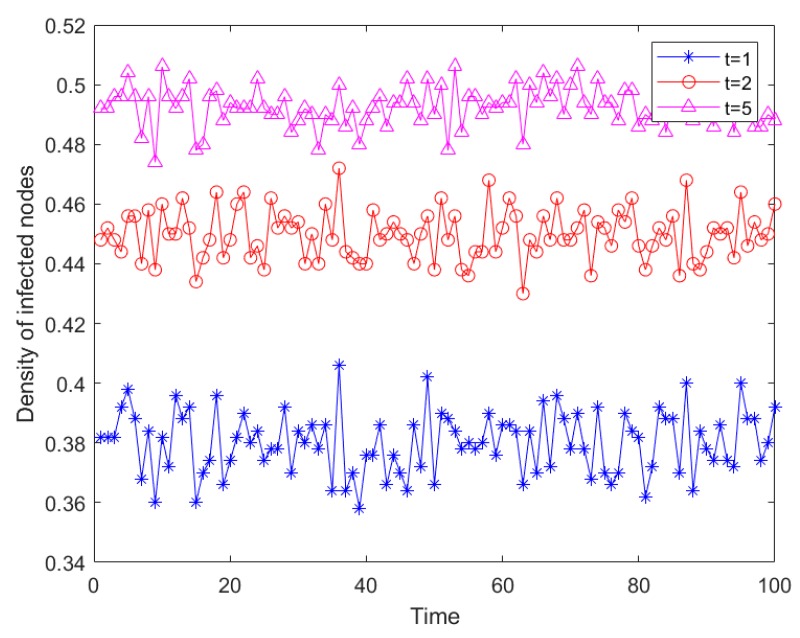
Risk propagation trends under different recovery times.

After a sudden disaster, rescue speed also affects the risk infection density. In order to explore the impact of rescue speed on risk infection density, the relevant parameters were set as follows: *N* = 500, the initial infection sources proportion was *Φ* = 0.1, the infection risk level was *α* = 0.5, the recovery capacity was *β* = 0.1, and the time was *t* = 100. In this experiment, the simulation time was extended so as to better compare the impact of different recovery speed on risk propagation and risk infection density under different recovery time. The detailed results are shown in Figure 11.

As shown in Figure 11, when the recovery time was taken as 1, 2, or 5, respectively, the risk infection density gradually increased. The speed of recovery time corresponded to the rescue speed in reality, and the increase in rescue speed effectively inhibited the risk propagation density. Compared with the recovery time from 1 to 2 and from 2 to 5, the former’s recovery time had a better inhibitory effect on risk propagation, and the fundamental reason for this lies in the “diminishing returns” effect in economics. In the actual emergency logistics rescue process, over-deployment of rescue may also lead to a decrease in emergency rescue efficiency and a decrease in the effect of controlling risk propagation.

From the above simulation results, it was discovered that in areas with high incidences of sudden disasters, due to the unpredictability and high disaster risks, the nodes in these regions had certain preventions and countermeasures for risks to reduce the damage degree to nodes in the regional network. In the process of emergency logistics risk control, enhancing the capacity of each node to deal with risk is of great significance. The capacity for acquiring rescue, the number of information communication channels, and adequate material support greatly reduces the risk of being infected. High risk must be considered, as it related to a stronger risk propagation capacity. The ability of risk propagation is determined by the long-term influence of nodes, therefore, the risk propagation between nodes should be taken into account during emergency logistics risk control, especially to strengthen the monitoring of key nodes. At the same risk level, the greater the importance of nodes is, the stronger the risk propagation ability is. Therefore, emergency disaster risk prevention mechanisms should be adopted for critical nodes in the region.

After the occurrence of a disaster, controlling the source risk can effectively reduce the spread of risk. According to the simulation results in this paper, reducing the proportion of risk infection sources significantly reduces the proportion of risk infection, which is especially important for controlling the risk propagation in the emergency logistics network. In addition, through the analysis of the evolution of risk propagation, the speed improvement of emergency logistics rescue reduces the risk infection density. However, attention should be paid to the efficiency of emergency logistics rescue deployment to reduce invalid rescues and improve emergency logistics rescue effectiveness.

## 5. Conclusions

In this paper, an emergency logistics network model based on material reliability was constructed to solve the risk propagation of emergency logistics firstly. Secondly, the evolution model of emergency logistics risk propagation was proposed. Thirdly, the influence factors of emergency logistics risk propagation were studied by using the improved SIS communication model, and the corresponding strategies for controlling emergency logistics risk propagation were also offered.

By simulating the evolution model of emergency logistics risk propagation, the following conclusions were made. (1) Material reliability had a great influence on emergency logistics risk propagation. The higher the material reliability was, the smaller the risk propagation scope was. (2) In the early stage of emergency logistics risk propagation, keeping the number of infected sources of emergency logistics risk low effectively reduced the proportion of emergency logistics risk infection. (3) In the process of risk propagation, the improvement of the recovery ability of nodes within the region has a greater impact on risk propagation than the improvement of risk infection level. Therefore, it was necessary to strengthen the reliability of its own material reservations, attach importance to external communication and ensure the accessibility of rescue channels. (4) After the occurrence of sudden disaster risk, the improvement of rescue speed had a good inhibitory effect on risk propagation. The effectiveness of rescue speed should be considered and excessive rescuing weakened the control effect on risk propagation and diffusion.

This paper mainly introduced the idea of risk propagation management to the field of emergency logistics, and some of the outcomes could also be applied to research in the medical field in the future, particularly in regard to infectious diseases that may occur when disasters occur. First of all, hospitals could estimate the influence scope of diseases according to the simulation results and then actively carry out relevant prevention and control measures so as to improve hospital management and reduce ineffective medical rescue resource use. Secondly, as patients in the disaster area, enough medical supplies should be prepared. Thirdly, policy makers could make targeted rescue policies based on the size of disasters, quickly control the spread of disasters, increase rescue speed, and reduce disaster losses.

Although this paper identified some valuable outcomes regarding the risk management of emergency logistics, there were still some limitations. In the future, the following aspects should be further explored:

(1) For the construction of emergency logistics network, this paper only studied the evolution mechanism of emergency logistics risk, without considering the impact of edge weight and node weight [28]. It is recommended to consider the heterogeneous characteristics of different demand nodes, apply the actual data to verify the validity of the model, and make it more realistic.

(2) In the process of emergency logistics risk propagation, risks may change in real time. Correspondingly, the prevention and control of risks should also be dynamic, and different demand nodes may have different methods to solve risks. Therefore, in the future, the perspective of risk dynamics should be explored so as to improve the ability of a whole region to deal with risks.

(3) Emergency logistics is a very complex system with different external environment interference factors, as well as internal coordination ones. As a result, several research efforts are necessary, such as evaluating the risk of emergency logistics, constructing reasonable risk assessment indicators of emergency logistics, optimizing assessment methods, grasping the risk of emergency logistics accurately, and improving response efficiency.

## Figures and Tables

**Table 1:** Symbol description.

***C_e_(i)***	Eigenvector centrality of node i
***ω_i_***	Risk propagation capacity of node i
***τ_i_***	Anti-risk capacity of node i
***F_i_***	Closeness centrality of of node i
**Q(λ_i_)**	0–1 variable, determine the risk propagation of node i
***O_i_***	Recovery capacity of node i after risk

**Table 2:** Topological characteristics of the emergency logistics network.

Average Path Length	Clustering Coefficient	Average Degree
2.665	0.026013	19.346
